# Pediatric Surgical Care in a Dutch Military Hospital in Afghanistan

**DOI:** 10.1007/s00268-015-3136-z

**Published:** 2015-07-09

**Authors:** Floris J. Idenburg, Thijs T. C. F. van Dongen, Edward C. T. H. Tan, Jaap H. Hamming, Luke P. H. Leenen, Rigo Hoencamp

**Affiliations:** Royal Netherlands Navy (R) and Division of Surgery, Department of Trauma Surgery, MC Haaglanden - Bronovo, Lijnbaan 32, 2512 VA Den Haag, The Netherlands; Division of Surgery, Department of Trauma, University Medical Centre Utrecht, Utrecht, The Netherlands; Royal Netherlands Army (R) and Division of Surgery, Department of Trauma Surgery, Radboud University Medical Centre, Nijmegen, The Netherlands; Department of Surgery, Leiden University Medical Centre, Leiden, The Netherlands

## Abstract

**Background:**

From August 2006–August 2010, as part of the ISAF mission, the Armed Forces of the Netherlands deployed a role 2 enhanced Medical Treatment Facility (R2E-MTF) to Uruzgan province, Afghanistan. Although from the principle doctrine not considered a primary task, care was delivered to civilians, including many children. Humanitarian aid accounted for a substantial part of the workload, necessitating medical, infrastructural, and logistical adaptations. Particularly pediatric care demanded specific expertise and equipment. In our pre-deployment preparations this aspect had been undervalued. Because these experiences could be influential in future mission planning, we analyzed our data and compared them with international reports.

**Methods:**

This is a retrospective, descriptive study. Using the hospital’s electronic database, all pediatric cases, defined as patients <17 years of age, who were admitted between August 2006 and August 2010 to the Dutch R2E-MTF at Multinational Base Tarin Kowt (MBTK), Urzugan, Afghanistan were analyzed.

**Results:**

Of the 2736 admissions, 415 (15.2 %) were pediatric. The majority (80.9 %, 336/415) of these admissions were for surgical, often trauma-related, pathology and required 610 surgical procedures, being 26 % of all procedures. Mean length of stay was 3.1 days. The male to female ratio was 70:30. Girls were significantly younger of age than boys. In-hospital mortality was 5.3 %.

**Conclusion:**

Pediatric patients made up a considerable part of the workload at the Dutch R2E-MTF in Uruzgan, Afghanistan. This is in line with other reports from the recent conflicts in Iraq and Afghanistan, but used definitions in reported series are inconsistent, making comparisons difficult. Our findings stress the need for a comprehensive, prospective, and coalition-wide patient registry with uniformly applied criteria. Civilian disaster and military operational planners should incorporate reported patient statistics in manning documents, future courses, training manuals, logistic planning, and doctrines, because pediatric care is a reality that cannot be ignored.

## Introduction

From 2003 to 2014, an International Security Assistance Force (ISAF) operated in Afghanistan under a peace enforcement mandate pursuant to Chapter VII of the UN Charter, authorized by the UN Security Council [1]. As part of ISAF and having Uruzgan province as its primary area of responsibility, The Kingdom of the Netherlands deployed Task Force Uruzgan (TFU) from August 2006–August 2010. A troubled and isolated province, Uruzgan in 2006 had a population of about 400,000. Access to medical care was extremely limited, with only a few basic health care facilities being operational, and Uruzgan Healthcare Performance Indicators revealing poor parameters. Initially there was no functioning provincial hospital. In those days, only two midwives were present and not a single surgeon [2]. Adhering to International Humanitarian Law and having signed the Geneva Conventions and the UN convention on the Rights of the Child, The Netherlands Armed Forces are obliged to take care of the civilian population during times of war, especially when local healthcare systems are non-existent. TFU and its Medical Treatment Facility were located at Multinational Base Tarin Kowt (MBTK), close to Uruzgans capital, Tarin Kowt. NATO’s medical doctrine is outlined in MC 326/2 NATO Medical Support Principles and Policies [3]. Within that doctrine, different levels of care are described. Treatment facilities are numbered, with higher numbers indicating progressive capabilities. TFU included a role 2 enhanced MTF (R2E-MTF), providing intermediate capability for triaging and treatment of patients, including damage control surgery (DCS), with two intensive care unit beds and a limited holding capacity. These abilities qualified this military medical facility as the best-equipped hospital of Uruzgan province. The primary mission of military medical treatment facilities is to provide care for wounded or sick service members. Treating civilians, including infants, children, and adolescents is not considered a core task for combat supporting hospitals. Taking care of children is sometimes even referred to as “mission creep” [4]. Medical Rules of Eligibility (MROE) limit admission of civilians to military medical treatment facilities to life, limb, or eyesight (LLE) threatening conditions. Yet, as part of an integral 3D approach, which includes defense, diplomacy, and development, striving for a stable post-conflict state, it is recognized as an opportunity to win the hearts and minds of the local population [5]. In 2006, our R2E-MTF was not adequately equipped to treat children and our staff was not specifically trained in pediatrics. Pre-deployment training programs and medical logistic supplies did not include pediatrics. But the facts in Afghanistan, with under-five infant mortality rates amongst the highest in the world [6], an estimated 49 % of the population being under 15 years of age [7], and children bearing the impacts of the armed conflict [8, 9], turned out different. The 4 years’ workload in Uruzgan included many pediatric cases [10], and adaptation was needed. The goals of this study are to determine the demographics and epidemiology of that population, and to compare our findings with other reports from Afghanistan and, if suitable and relevant, with Iraqi information. Results might influence future training, pre-deployment requirements, and logistics of the Dutch medical military forces.

## Methods

This descriptive, retrospective study was performed under a protocol for which approval was obtained from the Ministry of Defense (MOD) and the Institutional Review Board and Medical Ethical Committee of Leiden University, the Netherlands.

Using the hospitals electronic admission register, data collection and subsequent analysis of all pediatric cases, treated at the Dutch R2E-MTF between August 2006 and August 2010, were conducted. Pediatrics was defined as any patient under the age of 17. The association between two categorical variables was calculated by applying the Pearson *χ*^2^ test. In all cases, *p* < 0.05 was considered statistically significant. Statistical analyses were performed through a computerized software package: SPSS (Version 20, IBM Corporation, Armonk, New York). The categorical variables were analyzed by their absolute and relative frequencies in percentages.

## Results

The R2E-MTF database revealed a total of 2736 admissions in the studied period [10]. Of those, 2321 (84.8 %) were adults and 415 (15.2 %) infants and children. Soon after the hospital reached operational status, the first children were admitted, with admission rates remaining fairly constant over the years [2006 (Aug–Dec): 18, 2007: 103, 2008: 125, 2009: 107, 2010 (Jan–Jul): 62]. These patients were either self-referred by presenting themselves at the gate or referred by local health workers, non-governmental organizations, local elderly, the Provincial Reconstruction Team, or picked up by ISAF military personnel during patrols.

**Fig. 1:**
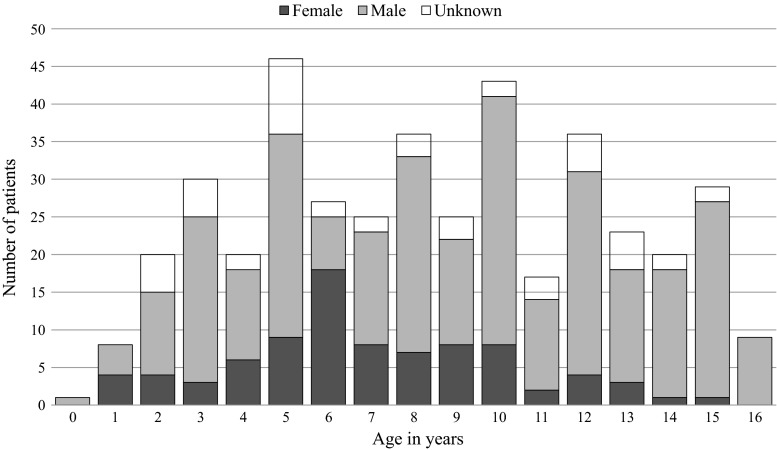
Distribution of pediatric admissions in age and sex (*n* = 415)

Of the admitted infants and children, 288 (69.4 %) were male and 76 (18.3 %) were female (*p* < 0.05). In 51 cases (12.3 %), we could not find sex documented. The age and sex distribution of the pediatric cases are represented in Fig. 1; the youngest child was 6 months of age. This pediatric population was registered as disease and non-battle injury (DNBI), and the delivered care considered humanitarian aid.

**Fig. 2:**
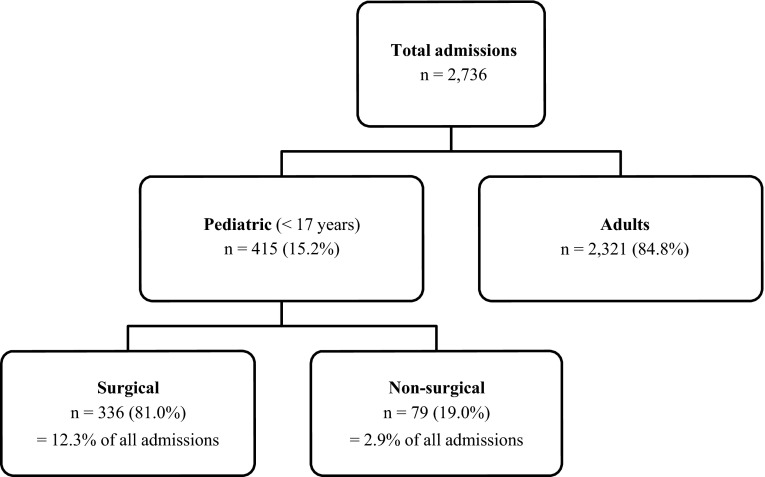
Distribution of admissions within the period 2006–2010. *n* indicates number

Counting only first-time admissions, 79 (19.1 %) children were admitted for a non-surgical diagnosis and 336 (80.9 %) had a surgery-requiring finding. From all admissions, 12.3 % were pediatric surgical (trauma and non-trauma) cases and 2.9 % were pediatric non-surgical (Fig. 2).

The mean length of stay was 3.1 days. The longest recorded admission, a burn patient, was 46 days.

The 336 pediatric surgical patients required a total of 610 surgical procedures, resulting in 1.8 operations per child. Details of the surgical procedures performed on pediatric cases are presented in Table 1.

**Table 1:** Surgical interventions on pediatric cases (*n* = 336)

Procedures performed	Number (%)
Head/neck	37 (6.1)
Thoracotomy	7 (1.1)
Chest drain	15 (2.5)
(DC) laparotomy	81 (13.3)
Genitals	25 (4.1)
Major amputation	17 (2.8)
Minor amputation finger/toe	6 (0.9)
Large arterial vessel	9 (1.4)
Extremity ORIF	57 (9.3)
Fasciotomy/escharotomy	14 (2.3)
External fixation	80 (13.1)
DID	164 (26.9)
DIS	28 (4.6)
Reconstruction/SSG	26 (4.3)
Minor general surgery	8 (1.3)
MUA	36 (5.9)
Total procedures	610 (100)

*DC* damage control, *ORIF* open reduction internal fixation, *DID* indicates debridement, irrigation, and dressing, *DIS* debridement, irrigation, and splinting, *SSG* split skin graft, *MUA* manipulation under anesthesia, *n* number

In the studied period, 2319 surgical procedures were performed on all admitted patients. From those, 796 were humanitarian, non-combat related, and performed on local nationals, leading to 26 % (610/2,319) of all registered surgical procedures, and 76 % (610/796) of all humanitarian surgical procedures, being pediatric.

**Fig. 3:**
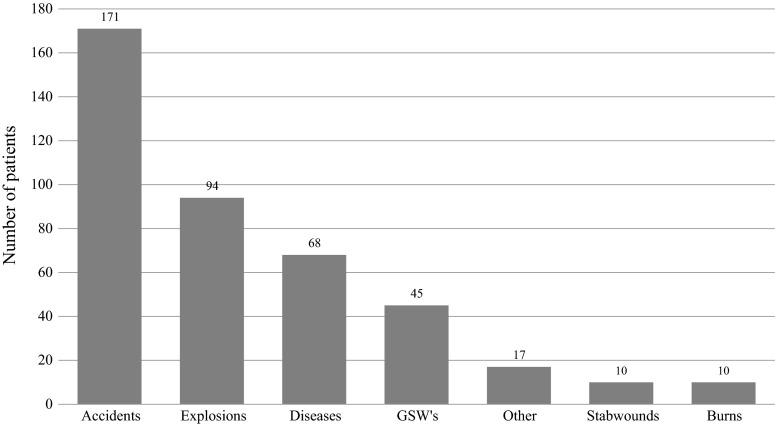
Causes for admission. GSW’s indicates gunshot wounds, and Others include intoxications and animal bites

The causes for admission in the studied population are shown in Fig. 3. Trauma admissions as a result of non-combat related incidents (like road traffic accidents, falls, dog bites, and near drowning) accounted for the majority (41.2 %) of cases, followed by explosions (22.7 %) and gunshot wounds (GSW) (10.8 %).

A wide variety of diseases, including pneumonia, malnutrition, meningitis, pyelonephritis, intoxications, snakebites, malignant-, congenital-, and developmental disorders accounted for 85 (20.4 %) admissions. Non-trauma surgical pathology incorporated abscesses, hernias, appendicitis, volvulus, and typhoid bowel perforations. Burns were the main cause for admission in ten patients, not separately counting burn wounds from explosions.

The discharge destination of the cases is presented in Table 2. The majority (60.5 %) of the children were discharged to home. Thirty-four children (8.2 %) needed further treatment and were referred to a higher echelon of care. Of the studied population, 22 (5.3 %) patients died. The known causes of death are summarized in Table 3.

**Table 2:** Discharge destination

Destination	Home	Local healthcare facility	Role 1	Role 3	Unknown	Deceased	Total
Number	251	95	3	34	10	22	415
Percentage	60.5	22.9	0.7	8.2	2.4	5.3	100

**Table 3:** Causes of death (*n* = 22)

Causes of death	Numbers
Abdominal DNBI	3
Blast injuries	8
Gunshot wounds	3
Pneumonia	1
Meningitis	2
Burns/multi organ failure	1
Accident	2
Sepsis	1
Multifactorial	1
Total	22

*DNBI* disease non-battle injury, *n* number

## Discussion

This study is the first report over a prolonged period from a single, static NATO role 2 MTF in Afghanistan. It aims to analyze the pediatric care delivered in the Dutch R2E-MTF in Uruzgan, Afghanistan (2006–2010), to compare our results with other reports, if possible with other Afghanistan related outcomes, and to learn lessons for future missions.

Our overall 15.2 % pediatric admission rate is lower than reported in earlier stages of the conflict [11, 12] but higher than in later stages [13, 14], possibly reflecting an increased security situation and effects of ISAF’s host nation capability and capacity building policy. Almost seventy percent of all pediatric admissions concerned a male infant. This male dominance reflects patterns in other reports [11, 15, 16]. Girls tend to be of younger age as well, which might be caused by boys being more out in the streets and therefore exposed to traffic or able to play with explosive remnants of war, or actively taking part in the conflict. It might, however, also reflect local referral tendencies and socio-cultural features, an experience also reported from Iraq [17].

Almost 20 % of all pediatric admissions were non-surgical. These patients were presented to us with a heterogeneous mixture of diseases and disabilities, including congenital syndromes. The relative small numbers do not lend themselves for statistical analysis or comparison. However, they stress the fact that in areas with underdeveloped or destroyed healthcare systems, military healthcare providers should be prepared for being confronted with a wide variety of childhood disorders.

The surgery-associated pediatric DNBI admissions resulted in 26 % of all surgical procedures being pediatric, which is higher than the 14.7, 10.8, and 6 % reported by others [14, 18, 19]. We could not identify one specific reason for this finding. Probably it reflects provincial demographics, local health care capabilities, and provincial reconstruction team efforts. The large majority (76 %) of all humanitarian (non-combat related) surgical procedures were performed on children. This underlines the reality that once a military medical treatment facility starts giving humanitarian assistance, its staff should anticipate on, and prepare for doing pediatric surgical procedures. Trauma-related interventions were the most common, which is in line with other reports. Blast injuries dominated over GSW, a trend reported by many others [15, 20–22].

Discharging this vulnerable patient population merits careful consideration. The majority could be discharged home. Comparing these findings with other hospitals in other regions is of no use. Discharge destinations are circumstantial and strongly influenced by patient requirements and locally available options. Due to the lack of proper local health care in Uruzgan, children could often not be discharged in an early phase. Over the years, the efforts of Provincial Reconstruction Teams resulted in the renewing of a local health care facility, known as TK-hospital. Ultimately 95 patients (22.9 %) could be transferred to that institute. Prolonged hospitalizations in this age group required adaptations in our standard operating instructions, like hosting family members and creating a pediatric ward. Initiating pediatric protocols, we started for example routine deworming of the children, introduced liquid antibiotics and adjusted our pain protocol. We did not deploy specialized caregivers [23]. As reported by others, for difficult cases, we had to rely on tele-medicine, consulting home-based specialists with modern means of communication [24].

Over the years, reported in-hospital mortality rates in military treatment facilities in Iraq and Afghanistan were fairly constant and varied roughly between 3 and 9 %. Our in-hospital mortality rate of 5.3 % is comparable with the literature [4, 12, 15, 17, 19–21, 25–29]. Sometimes the extend of the injuries or the severity of the disease was incompatible with life and care had to be withdrawn. Severe neurological damage, multi organ failure, extensive burns, and irreversible sepsis contributed to these decisions.

Warzone pediatric trauma statistics differ from civilian figures in numbers and in mechanisms of injury. Every armed conflict takes its toll on the civilian population. This phenomenon of unintentional or incidental damage as a result of military action against targeted enemy forces or facilities, is referred to as collateral damage [30]. One of the most striking examples dates back to Vietnam, June 8th, 1972; a 9-year-old, naked, severely burned girl, fleeing her village after a napalm attack. Pictures became emblematic of the human suffering that war causes. The devastating effects that war has on children, have been described extensively [31]. Characterized by unclear frontiers between soldiers and civilians, the conflict in Afghanistan is referred to as an internationalized civil war [32]. Large-scale pediatric, humanitarian care by combat-deployed military medical units is a relative new trend and likely the result of the different duration and characteristics of modern-time conflicts. Reports of humanitarian pediatric care during active hostilities in military hospitals during the Korean War are scarce and anecdotal. During the Vietnam conflict, with a rising number of civilian casualties, complete emergency care for civilians was authorized. As part of Medical Civic Action Programs (MEDCAPS) children were treated, but we could not find any reported series. In Iraq and Afghanistan, MTF’s were static, semi-permanent units, fully equipped, often the best and frequently the only functioning health care facility in their region, not aiming to, but de facto replacing or augmenting local health care. Hence, the civilian population, including infants and children, called upon the medical staff not only in LLE situations, but also for elective general surgical care, infections, and non-surgical diseases. Since 2003, several reports regarding this phenomenon have been published [4, 20, 33, 34], addressing the medical, logistical, and ethical challenges that field hospitals face when it comes to pediatric care.

Our study has several limitations. It is retrospective in nature, based on often incomplete data, and it lacks systematic follow-up. Although patients were discharged with an appointment at our follow-up clinic, many did never show up, probably due to long travel distances, lacking means of transportation, local habits, and nature of the conflict.

When comparing our findings with earlier reports, several difficulties are encountered. First of all, findings are biased by a striking lack of uniformity in the used age definition of pediatric patients. It ranges from not specifically reported [14] to under 15 [13, 20, 35], under 16 [15, 16, 21], under 17 [12], under 18 [4, 17, 25–29], and under 19 years of age [11]. Reasons are generally not discussed, but include “patients older than 14 years were often combatants” [20]. Interestingly, some co-authors are even not consistent in their age definition in consecutive publications [11, 17, 26, 28, 35]. Besides, reliable means of identification, with trustworthy dates of birth, are almost non-existent in Afghanistan. This fact has been reported earlier, with up to 48 % non-documented ages [15, 16]. Due to differences in inclusion and exclusion criteria, data extraction, and outcomes of interest, a statistical test for heterogeneity (ea. *I*^2^ test) is not suitable to evaluate these differences. We based our study on the age as documented in the medical records and arbitrarily defined our study population as younger than 17 years. We felt that care for patients above the age of 16 progressively resembled our adult care and that including this age group might overemphasize the professional, medical, logistical, and operational issues that prompted this study. In pediatric literature, however, the full implications of pediatric age groups for health care and research are not completely understood and under debate [36]. For future research, a universally accepted cut-off age is a precondition to make reliable comparisons. Realizing that treating diseases, malnourishment and developmental disorders might require a different cut-off age than trauma care and that any definition will be subject to debate, we suggest that in military research, pediatric care is arbitrarily defined as care given to patients under the age of 17.

Secondly, most reports come from combat supporting hospitals (Table 4). Those patient characteristics and statistics [13–15, 18] are potentially influenced by work done at forward located installations like combat outpost’s, forward operating bases, forward surgical teams [11], and role 1 and 2 MTF’s. It is perceivable that non-surgical, medical aid is more often provided in an outpatient setting at these installations. Consequently, surgical cases might dominate in role 3 reports [20, 35].

**Table 4:** Reports on pediatric care in military hospitals in Iraq and Afghanistan

Author	Specific pediatric	Study period	Afgh.	Iraq	Hosp	Age	Set up	All admissions	Trauma admissions	All surgical cases	Blast injury cases	ICU cases	Total admissions (*n*)	% Ped of all admissions	Ped cases (*n*)	% of Ped non-trauma	Ped mortality rate
	Number of patients																
Gurney [16]	Yes	Mar 03–May 03		+	Role 3	<16	R	+					2720	3.4	78	16.7	NR
Beekley [11]	No	Aug 02–Mar 03	+		FST	<19	R	+					90	22	20	NR	NR
Beitler [12]	No	Dec 02–Jun 03	+		CSH	<17	R	+					477	18.8	90	35.5	3.3
Coppola [4]	Yes	Jan 04–May 05		+	Role 3	<18	R	+					1626	5.2	85	44	5.9
McGuigan [28]	Yes	Jan 04–Dec 04		+	CSH	<18	R		+				3293	3	99	NA	9
Burnett [26]	Yes	Dec 01–Dec 04	+	+	FST/CSH	<18	R	+					24,227	4.2	1012	18	5.8
Spinella [29]	Yes	Dec 01–May 07	+	+	CSH	<18	R		+				1305	7.1	1305	21	5.4
Creamer [17]	Yes	Jan 02–Oct 07	+	+	CSH	<18	R	+					20,000	10	2060	25	6.9
Harris [27]	Yes	Jul 08–Sep 08	+		Role 3	<18	R					+	NA	NA	15	6.7	6.7
Nordmann [19]	Yes	NR	+		Role 3	–	R						NA	NA	31	NR	3.2
Borgman [25]	Yes	2001–2011	+	+	FST/CSH	<18	R	+					128,582	5.8	7505	21	8.5
Beckett [13]	No	Oct 09–Dec 10	+		Role 3	<15	R	+					2599	5.5	197	NA	NR
Jacobs [14]	No	Nov 08–Nov 10	+		Role 3	NR	R			+			4276	6	299	NA	NR
Arul [15]	Yes	Jan 11–Apr 11	+		Role 3	<16	P	+					NR	NR	82	6	2.5
Wilson [22]	Yes	Jun 10–Mar 11	+		CSH	< 18	R		+				406	10	41	NR	NR
Edwards [35]	Yes	2002–2010	+	+	Role 3	<15	R				+		4928	24.6	1213	NA	NR
Edwards [20]	Yes	2002–2012	+		Role 3	<15	R	+					NR	NA	3292	28	5–8
Edwards [20]	Yes	2002–2012		+	Role 3	<15	R	+					NR	NA	2981	19	5–11
Mckechnie [21]	Yes	Jul 08–Nov 12	+		Role 3	<16	R		+				NR	NR	766	NA	8
Brondex [33]	No	Jun 12–Dec 12	+		Role 3	<16	P	+					439	13.4	59	NR	NR
Our study 2014	Yes	Aug 06–Aug 10	+		Role 2	<17	R	+					2736	15.2	415	19.1	5.3

*Afgh* Afghanistan, *Hosp* type of hospital, *ICU* intensive care unit, *Ped* pediatrics, *FST* Forward Surgical Team, *CSH* combat support hospital, *n* number, *NR* not reported, *NA* not applicable, *R* retrospective, *P* prospective

Thirdly, inclusion criteria differ from only trauma admissions [15, 21, 25], to all admissions [11, 17], all surgical cases, including caesarean sections [14, 18], only trauma-related surgical cases [21], only blast trauma [35], or even a small subgroup of intensive care cases [27].

Fourthly, reports vary significantly in the studied time-span, ranging from 37 days [16] to almost 11 years [20, 35], with sizes of the studied population ranging from 20 in single hospital experiences [11] to multi-hospital reports including 7505 patients [25]. Results are therefore likely influenced by seasonality, troop surges, different stages of the conflicts with corresponding intensity of hostilities, and the inherently varying interpretations of the rules of eligibility.

Finally, some studies limit their experiences to either Iraq [4, 16, 25, 28] or Afghanistan [11–15, 21, 27], while others include both countries [17, 20, 26, 29, 35]. This might have influenced findings, due to the distinctive characteristics of both countries, including substantial differences in existing local healthcare facilities and fundamental dissimilarities in the respective conflict scenarios.

## Conclusion

Pediatric care is by doctrine, not considered a task of a military healthcare facility in a combat zone. The reality of the last 10 years in Iraq and Afghanistan, however, was different. Pediatric admission rates fluctuated over the years, ranging from 3 % to as high as 18 %, but are influenced by the used age definitions. Tens of thousands of sick, malnourished, and injured children were treated. This work, done at all levels of the military medical support organization, was challenging. Outcomes were often successful, but sometimes our efforts were in vain. Reported in-hospital mortality rates varied roughly from 3 to 9 %. Witnessing children succumbing to their diseases or injuries can be very confronting, but, generally, pediatric humanitarian work is rewarding.

Our findings stress the need for the implementation of a coalition-wide prospective database, with clear and uniformly applied definitions. Till then, making comparisons and drawing reliable conclusions will be difficult. But in modern-time conflicts, delivering pediatric care is to be considered a structural and inevitable aspect of military healthcare in a combat zone. Future conflicts will have their own, distinctive dynamics, requiring swift adaptations in current training programs, staffing schedules, logistic processes, and in updating training manuals. Pre-deployment training for specialists and general duty medical officers should include a dedicated course like the Pediatric Advanced Life Support (PALS), or existing pre-deployment courses that address the unique aspects of pediatric care, like Surgical Training in Austere Environments (STAE) as offered by the Royal College of Surgeons [37] or the Joint Forces Combat Trauma Management Course (JFCTMC) [38]. Not  only for military operations, but also in preparing for disaster response.
